# *Streptococcus pneumoniae* disrupts pulmonary immune defence via elastase release following pneumolysin-dependent neutrophil lysis

**DOI:** 10.1038/srep38013

**Published:** 2016-11-28

**Authors:** Hisanori Domon, Masataka Oda, Tomoki Maekawa, Kosuke Nagai, Wataru Takeda, Yutaka Terao

**Affiliations:** 1 Division of Microbiology and Infectious Diseases, Niigata University Graduate School of Medical and Dental Sciences, Chuo-ku, Niigata, Japan; 2 Research Center for Advanced Oral Science, Niigata University, Graduate School of Medical and Dental Sciences, Chuo-ku, Niigata, Japan; 3 Faculty of Dentistry, Niigata University, Chuo-ku, Niigata, Japan

## Abstract

*Streptococcus pneumoniae* is a leading cause of bacterial pneumonia and is the principal cause of morbidity and mortality worldwide. Previous studies suggested that excessive activation of neutrophils results in the release of neutrophil elastase, which contributes to lung injury in severe pneumonia. Although both pneumococcal virulence factors and neutrophil elastase contribute to the development and progression of pneumonia, there are no studies analysing relationships between these factors. Here, we showed that pneumolysin, a pneumococcal pore-forming toxin, induced cell lysis in primary isolated human neutrophils, leading to the release of neutrophil elastase. Pneumolysin exerted minimal cytotoxicity against alveolar epithelial cells and macrophages, whereas neutrophil elastase induced detachment of alveolar epithelial cells and impaired phagocytic activity in macrophages. Additionally, activation of neutrophil elastase did not exert bactericidal activity against *S. pneumoniae in vitro*. P2X_7_ receptor, which belongs to a family of purinergic receptors, was involved in pneumolysin-induced cell lysis. These findings suggested that infiltrated neutrophils are the primary target cells of pneumolysin, and that *S. pneumoniae* exploits neutrophil-elastase leakage to induce the disruption of pulmonary immune defences, thereby causing lung injury.

*Streptococcus pneumoniae*, which is also called pneumococcus, is a Gram-positive Diplococcus and a major human pathogen. This bacterium causes bacterial pneumonia, meningitis, otitis media, and septicaemia, and is the principal cause of morbidity and mortality in young children, elderly people, and patients with immunodeficiencies^1^. The primary treatment of pneumococcal diseases involves the use of effective antibiotics; however, with the widespread availability and overuse of antibiotics, multidrug-resistant strains of pneumococci have become increasingly prevalent^2^, with 30% of *S. pneumoniae* in pneumococcal diseases fully resistant to one or more antibiotics. Additionally, total medical costs associated with pneumococcal pneumonia are >$96 million per year in the United States^3^. Therefore, basic research is still necessary to provide additional therapeutic targets to prevent and treat pneumococcal pneumonia.

A variety of pneumococcal virulence factors, including pneumolysin (PLY) and the autolytic enzyme LytA, contribute to the development of pneumococcal diseases^4^. PLY is a potent intracellular toxin that causes inflammation and tissue damage and varies little with serotype. PLY toxicity is associated with its ability to induce ring-shaped pores in cholesterol-containing membranes that mediate cell death^5^, whereas LytA is responsible for the characteristic autolytic behaviour associated with pneumococcus. It was reported that LytA potentially contributes to pneumococcal pathogenesis by catalysing the release of intracellular toxins, such as PLY, and generating pro-inflammatory cell-wall fragments^6^,^7^.

The immune response to pneumococcal pneumonia is generally characterized as an intense inflammatory reaction induced mainly by cell-wall components of the bacterium^8^. Recognition of the bacterium by pattern-recognition receptors located on the cell surface of lung epithelium and alveolar macrophages activates the induction of pro-inflammatory cytokines and chemokines, leading to rapid and heavy infiltration of neutrophils into infected lungs^9^. Although neutrophils are one of the first lines of defence against microbial pathogens, it was recently reported that excessive neutrophil activation caused the release of elastase, which contributed to lung injury in pneumococcal pneumonia^10^,^11^.

Neutrophil elastase (NE) is a potent serine protease that acts as a powerful host-defence component and exerts antimicrobial activity against a wide variety of bacteria, including *S. pneumoniae*, which is phagocytosed by neutrophils^12^. However, NE degrades a variety of extracellular-matrix proteins^13^, as well as lung-surfactant proteins^14^, which act as important host-defence agents against lung infections. Therefore, excessive release of NE can damage surrounding tissues and contribute to lung dysfunction associated with pneumonia. Consequently, patients with severe pneumonia exhibit increased levels of NE in lung^15^ and plasma^16^, and in animal models, intratracheal inoculation of *S. pneumoniae* causes acute lung injury characterized by increased accumulation of neutrophils and elevation of NE activity in bronchoalveolar-lavage fluid^17^,^18^. However, the underlying mechanisms by which neutrophils release NE in response to *S. pneumoniae* infection remain elusive.

Although both pneumococcal virulence factors and NE contribute to the development and progression of pneumococcal pneumonia and lung injury, respectively, there are few studies analysing the relationship between these factors, which eventually damage lung cells. In this study, we hypothesized that pneumococcal PLY induces neutrophil cell death and subsequent release of NE in an autolysis-dependent manner. To test this hypothesis, we examined the cytotoxicity of pneumococcal-culture supernatant or PLY on primary isolated human neutrophils and investigated the sensitivity of alveolar epithelial cells and macrophages to PLY.

## Results

### *S. pneumoniae* induces cytotoxicity against human neutrophils in an autolysis-dependent manner

**Figure 1:**
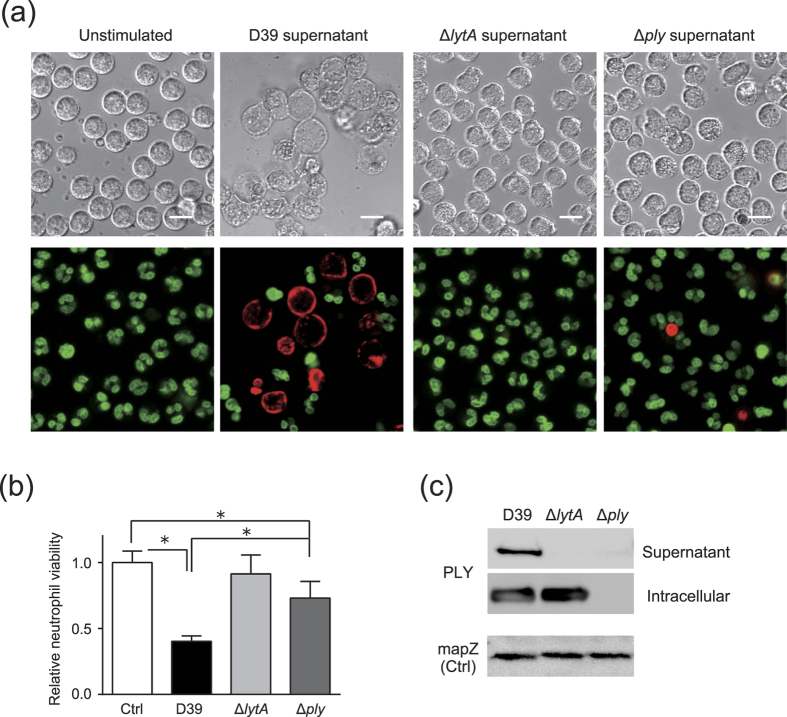
*Streptococcus pneumoniae* culture supernatant induces cytotoxicity against human neutrophils in an autolysis-dependent manner. Human neutrophils isolated from peripheral blood were exposed for 8 h to supernatant from *S. pneumoniae* wild-type strain D39, Δ*lytA*, or Δ*ply* cultures. (**a**) Representative differential interference contrast images and fluorescence images of cells stained by Hoechst 33342 (DNA; green) and ethidium homodimer III (necrotic cells; red) are shown. Scale bar: 10 μm. (**b**) Cell-viability assay was performed to evaluate supernatant cytotoxicity. Data represent the mean ± SD of quadruplicate experiments and were evaluated using one-way analysis of variance with Tukey’s multiple-comparisons test. *p < 0.05 between indicated groups. (**c**) Intracellular and extracellular (culture supernatant) expression of PLY protein in D39, Δ*lytA*, and Δ*ply* was determined by western blot analysis. mapZ, pneumococcal mid-cell-anchored protein Z; PLY, pneumolysin; SD, standard deviation.

We first investigated whether pneumococcal-culture supernatant can induce cytotoxicity in human neutrophils. Figure 1a shows that treatment with culture supernatant from an *S. pneumoniae* wild-type strain D39 induced cell lysis against neutrophils, and cell-viability assays also indicated significant cytotoxicity induced by treatment with the culture supernatant (Fig. 1b). We next examined whether the culture supernatant from *lytA*-isogenic mutant (Δ*lytA*) and *ply*-isogenic mutant (Δ*ply*) strains induced cytotoxicity against neutrophils. Interestingly, treatment with Δ*lytA*-culture supernatant did not induce significant levels of cytotoxicity (Fig. 1a and b), and, although treatment with Δ*ply*-culture supernatant slightly decreased the number of viable neutrophils, the level of induced cytotoxicity was significantly lower than that induced by treatment with *S. pneumoniae* wild-type strain culture supernatant. These findings suggested that LytA or PLY expression induced neutrophil cell death. Western blot analysis demonstrated that PLY was detected in *S. pneumoniae* wild-type strain culture supernatant, whereas it was not detected in the supernatant of Δ*lytA* or Δ*ply* strains (Fig. 1c), suggesting that PLY release from *S. pneumoniae* is associated with its autolysis. When PLY level was analysed by densitometry, PLY protein level in the *S. pneumoniae* wild-type strain culture supernatant was 6.1 ± 0.4 μg/mL (Fig. S1).

### Neutrophils are more susceptible to PLY-induced cell death than A549 and RAW264.7 cells

**Figure 2:**
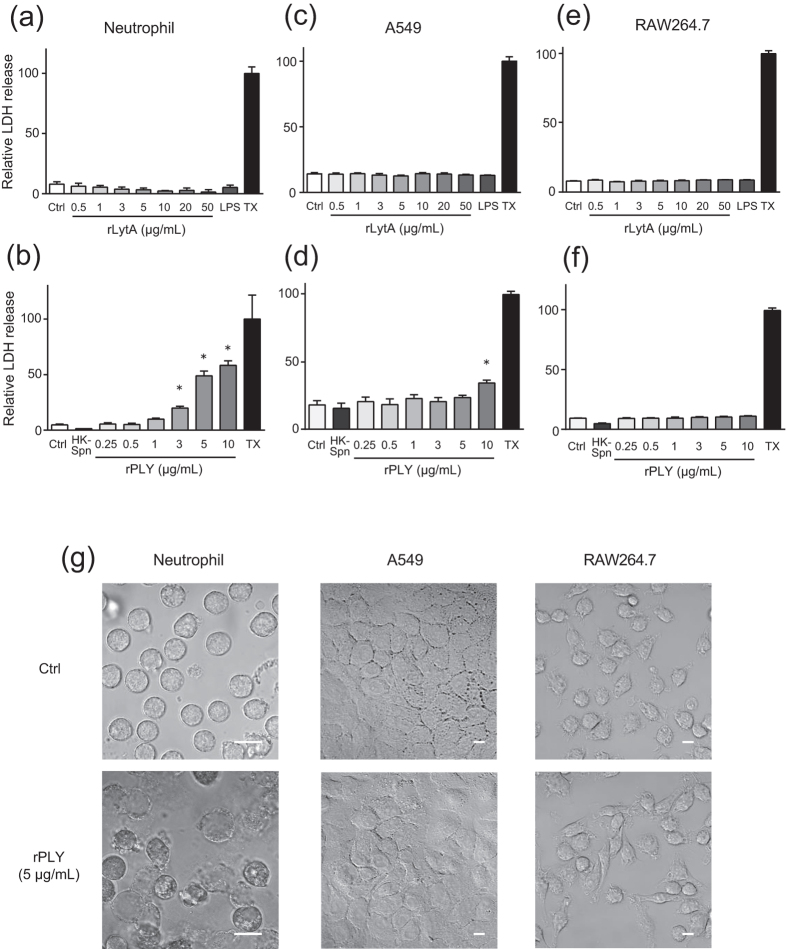
Neutrophils are susceptible to rPLY-induced cell death, whereas A549 and RAW264.7 cells are resistant. (**a,b**) Human neutrophils, (**c,d**) A549 alveolar epithelial cells, or (**e,f**) RAW264.7 macrophages were exposed to various concentration of rLytA (0.5–50 μg/mL), LPS (100 ng/mL) (**a,c,e**), rPLY (0.25–10 μg/mL), or heat-killed *Streptococcus pneumoniae* D39 (HK-Spn) (**b,d,f**) for 6 h, followed by evaluation of cytotoxicity by LDH assay. Data represent the mean ± SD of quadruplicate experiments and were evaluated using one-way analysis of variance with Dunnett’s multiple-comparisons test. *Significantly different from the control group at *p* < 0.05. (**g**) Representative microscopic image of rPLY-treated (5 μg/mL) cells. Scale bar: 10 μm. LDH, lactate dehydrogenase; LPS, lipopolysaccharide; rPLY, recombinant pneumolysin; SD, standard deviation; TX, Triton X-100.

We next investigated whether LytA or PLY induces cytotoxicity against neutrophils. Administration of recombinant PLY (rPLY) caused lactate dehydrogenase (LDH) leakage from neutrophils in a dose-dependent manner, which was not observed following administration of recombinant LytA (rLytA) (Fig. 2a and b). We also examined the cytotoxicity of these proteins against A549 human lung epithelial cells and RAW264.7 mouse macrophages. As shown in Fig. 2c–f, rLytA did not exhibit cytotoxicity against these cells, and rPLY did so only at high concentrations (10 μg/mL), which induced low levels of LDH leakage from A549 cells. In addition, cell viability assay showed consistent results (Fig. S2). Microscopic analysis also revealed that rPLY administration induced cell lysis of neutrophils, while it did not induce morphological changes in A549 or RAW264.7 cells (Fig. 2g). These findings suggested that autolysis-induced release of PLY selectively eliminated neutrophils.

### Neutrophils release NE in response to rPLY stimulation

**Figure 3:**
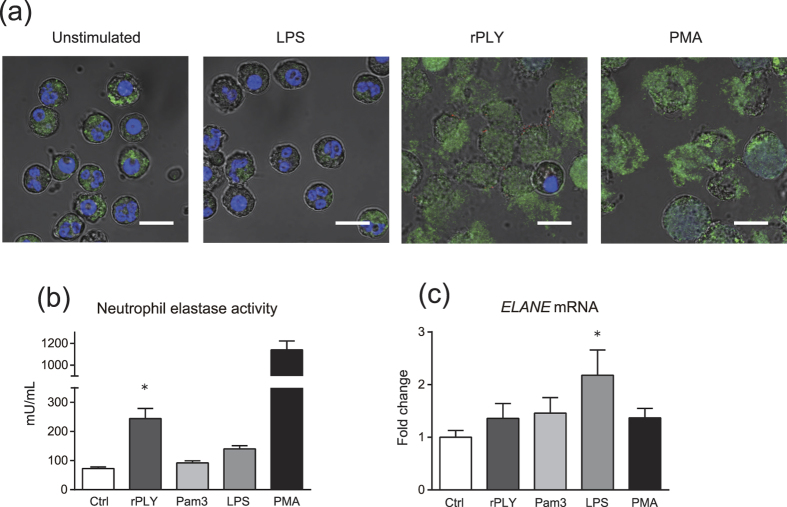
Neutrophils release NE in response to rPLY stimulation. Human neutrophils (5 × 10^6^ cells/200 μL) were exposed to rPLY (5 μg/mL), Pam3CSK4 (1 μg/mL), LPS (100 ng/mL), or PMA (40 nM) for 6 h. (**a**) Representative fluorescence-microscopy images of neutrophils stained for DNA (DAPI; blue), NE (green), and rPLY (red). Scale bar: 10 μm. (**b**) NE activity in the culture supernatant was evaluated using an elastase-activity assay kit. (**c**) Real-time PCR was performed to quantify NE (*ELANE*) mRNA in neutrophils exposed to these stimuli for 3 h. The relative quantity of *ELANE* mRNA was normalized to the relative quantity of *GAPDH* mRNA. (**b,c**) Data represent the mean ± SD of quadruplicate experiments and were evaluated using one-way analysis of variance with Dunnett’s multiple-comparisons test. *Significantly different from the control group at *p* < 0.05. LDH, lactate dehydrogenase; NE, neutrophil elastase; rPLY, recombinant pneumolysin; SD, standard deviation.

We hypothesized that rPLY-induced neutrophil cell death results in the release of NE and leads to lung injury. To test this hypothesis during acute inflammation *in vivo*, we increased neutrophil density in our *in vitro* assays to values typically accumulated in inflamed tissue (5 × 10^6^ neutrophils/200 μL)^19^. Immunofluorescence confocal microscopy analyses showed that rPLY-treated neutrophils released NE (Fig. 3a). Consistently, rPLY stimulation significantly increased NE activity in neutrophil-culture supernatant, whereas toll-like receptor (TLR) agonists had little effect (Fig. 3b). However, rPLY treatment did not elicit upregulation in *ELANE* mRNA transcription, suggesting that rPLY-induced cell lysis resulted in NE release without promoting NE-gene transcription (Fig. 3c).

### NE induces A549-cell detachment

**Figure 4:**
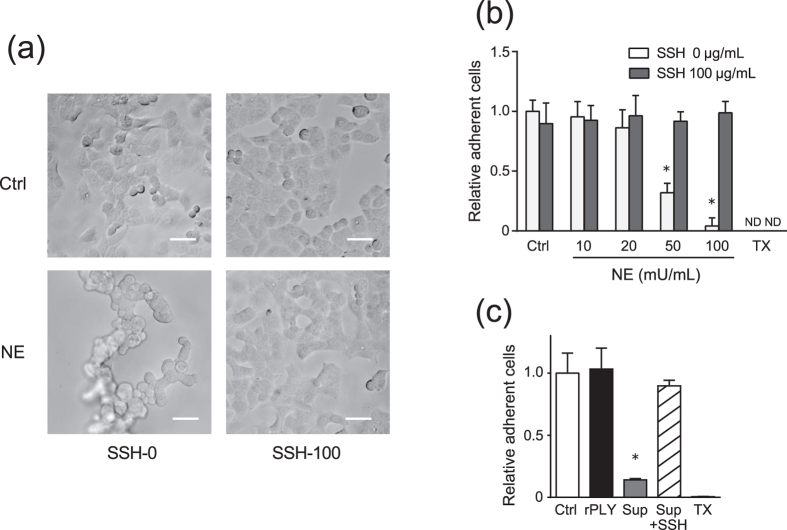
NE induces A549-cell detachment. A549 cells were cultured in serum-free DMEM and exposed to various concentrations (10–100 mU/mL) of NE in the presence or absence of the NE inhibitor sivelestat (100 μg/mL) for 6 h. (**a**) Morphologic changes in cells exposed to 100 mU/mL NE were observed by optical microscope. Scale bar: 100 μm. (**b**) MTT assay was performed to quantify A549-cell detachment. (**c**) A549 cells were exposed to supernatant (Sup) from rPLY-treated human neutrophils for 3 h, followed by MTT assay to quantify epithelial cell detachment. (**b,c**) Data represent the mean ± SD of quadruplicate experiments and were evaluated using one-way analysis of variance with Dunnett’s multiple-comparisons test. *Significantly different from the control group at *p* < 0.05. MTT, thiazolyl blue tetrazolium bromide; ND, not detected; NE, neutrophil elastase; rPLY, recombinant pneumolysin; SD, standard deviation; SSH-0, sivelestat sodium hydrate 0 μg/mL, SSH-100, sivelestat sodium hydrate 100 μg/mL.

Our observations prompted us to further examine whether NE release induced cellular dysfunction in alveolar epithelial cells and macrophages, both of which play important roles in antimicrobial defence against pneumonia^20^,^21^. As shown in Fig. 4a and b, NE release induced A549-cell detachment within 6 h in a dose-dependent manner. Additionally, treatment with the culture supernatant from rPLY-treated neutrophils also induced A549-cell detachment, whereas rPLY treatment alone did not affect cell detachment (Fig. 4c). Furthermore, this effect was counteracted by administration of the NE inhibitor sivelestat (Fig. 4a–c).

Previous studies indicated that NE treatment elicited upregulation of interleukin (IL) 8 mRNA transcription in alveolar epithelial cells^22^,^23^; however, our results showed that IL-8-protein levels were not elevated in the supernatant of NE-treated A549 cells (Fig. S3). These results may be due to IL-8 degradation by NE under serum-free conditions (Fig. S4).

### NE induces RAW264.7-cell detachment and impairs phagocytic activity

**Figure 5:**
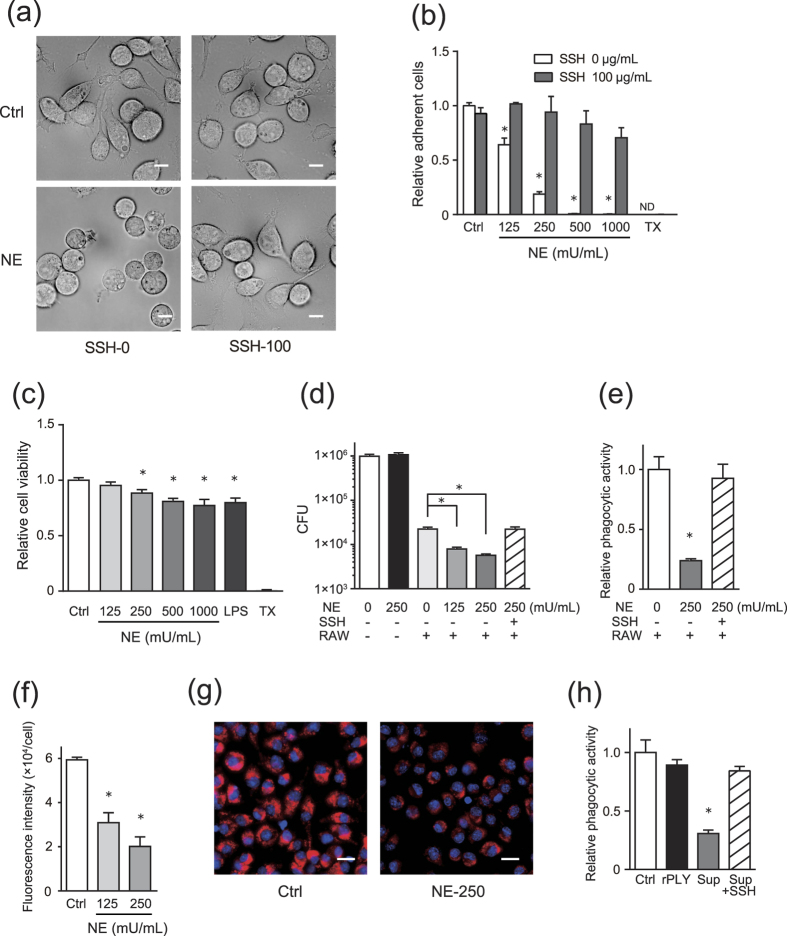
NE induces detachment of RAW264.7 cells and impairs phagocytic activity. RAW264.7 cells were cultured in serum-free DMEM and exposed to various concentrations (125–1000 mU/mL) of NE in the presence or absence of the NE inhibitor sivelestat (100 μg/mL) for 6 h. (**a**) Morphologic changes in cells were observed by optical microscope. Scale bar: 10 μm. (**b**) MTT assay was performed to quantify macrophage detachment. (**c**) Cell-viability assays. (**d,e**) NE-treated RAW264.7 cells were infected with *Streptococcus pneumoniae* D39 (MOI = 10) for 1 h, and (**d**) viable counts of phagocytosed *S. pneumoniae* were determined by plating serially diluted macrophage lysates on blood-agar plates. (**e**) Relative phagocytic activity of NE-treated RAW264.7 cells compared to untreated cells against *S. pneumoniae*. (**f,g**) NE-treated RAW264.7 cells were incubated in DMEM containing pHrodo red *Staphylococcus aureus* bioparticles for 30 min, and (**f**) the fluorescence intensity per cell was calculated. (**g**) Representative fluorescence-microscopy images of cells and nuclei stained with the bioparticles (red) and DAPI (blue), respectively. (**h**) RAW264.7 cells were exposed to culture supernatant (Sup) from rPLY-treated neutrophils or rPLY (5 μg/mL) for 3 h, followed by infection with *S. pneumoniae* D39 (MOI = 10) for 1 h and measurement of relative phagocytic activity. (**b–f,h**) Data represent the mean ± SD of quadruplicate experiments and were evaluated using one-way analysis of variance with Dunnett’s multiple-comparisons test. *Significantly different from the control groups at *p* < 0.05. MTT, thiazolyl blue tetrazolium bromide; ND, not detected; NE, neutrophil elastase; rPLY, recombinant pneumolysin; SD, standard deviation; SSH-0, sivelestat sodium hydrate 0 μg/mL, SSH-100, sivelestat sodium hydrate 100 μg/mL.

We next examined whether NE can induce macrophage detachment. Although NE treatment caused cell rounding and induced RAW264.7-cell detachment (Fig. 5a and b), 90% of the cells remained viable in Dulbecco’s modified Eagle’s medium (DMEM) containing 250 mU/mL NE (Fig. 5c). Therefore, we assessed the effect of NE on phagocytic activity in RAW264.7 cells. Interestingly, pre-treatment of the cells with NE for 6 h significantly decreased phagocytosed *S. pneumoniae* in RAW264.7 cells (Fig. 5d and e). We also measured phagocytic activity based on the uptake of indicators in NE-treated cells according to fluorescence intensity. The fluorescence intensity in NE-treated cells was significantly lower than that observed in control cells after a 30-min incubation with phagocytosis indicators (Fig. 5f and g). Additionally, administration of culture supernatant from rPLY-treated neutrophils also impaired phagocytic activity (Fig. 5h). Interestingly, this effect were counteracted by sivelestat treatment (Fig. 5a,b,d,e and h).

### NE did not exhibit bactericidal activity against *S. pneumoniae*

**Figure 6:**
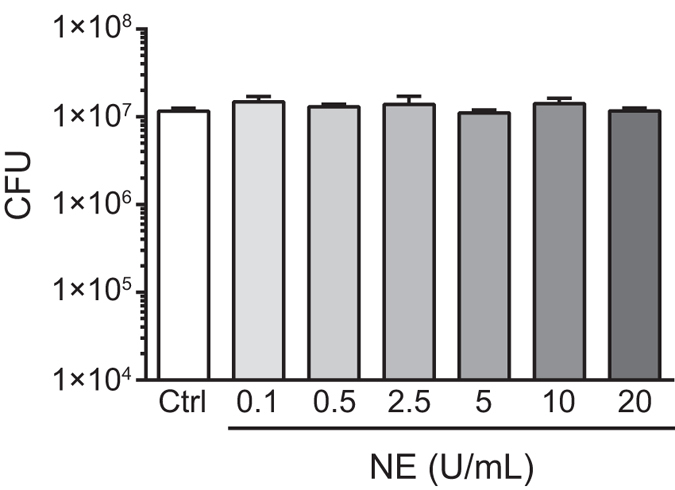
NE does not exhibit bactericidal activity against *Streptococcus pneumoniae in vitro.* *S. pneumoniae* D39 was cultured in TS broth until reaching the exponential-growth phase, followed by exposure to various concentrations (0.1–20 U/mL) of NE for 6 h, dilution, and inoculation onto blood-agar plates. Plates were incubated under aerobic condition at 37 °C for 2 to 3 days. Data represent the mean ± SD of quadruplicate experiments. NE, neutrophil elastase; SD, standard deviation; TS, tryptic soy.

It has been reported that NE plays an important role in intracellular bacterial killing by neutrophils^12^. Here, we examined whether NE exerted bactericidal activity against *S. pneumoniae* and observed that NE did not decrease the number of viable *S. pneumoniae in vitro* (Fig. 6), suggesting that rPLY-induced NE release did not adversely affect *S. pneumoniae* viability.

### P2X_7_ receptor is a possible membrane target of PLY

**Figure 7:**
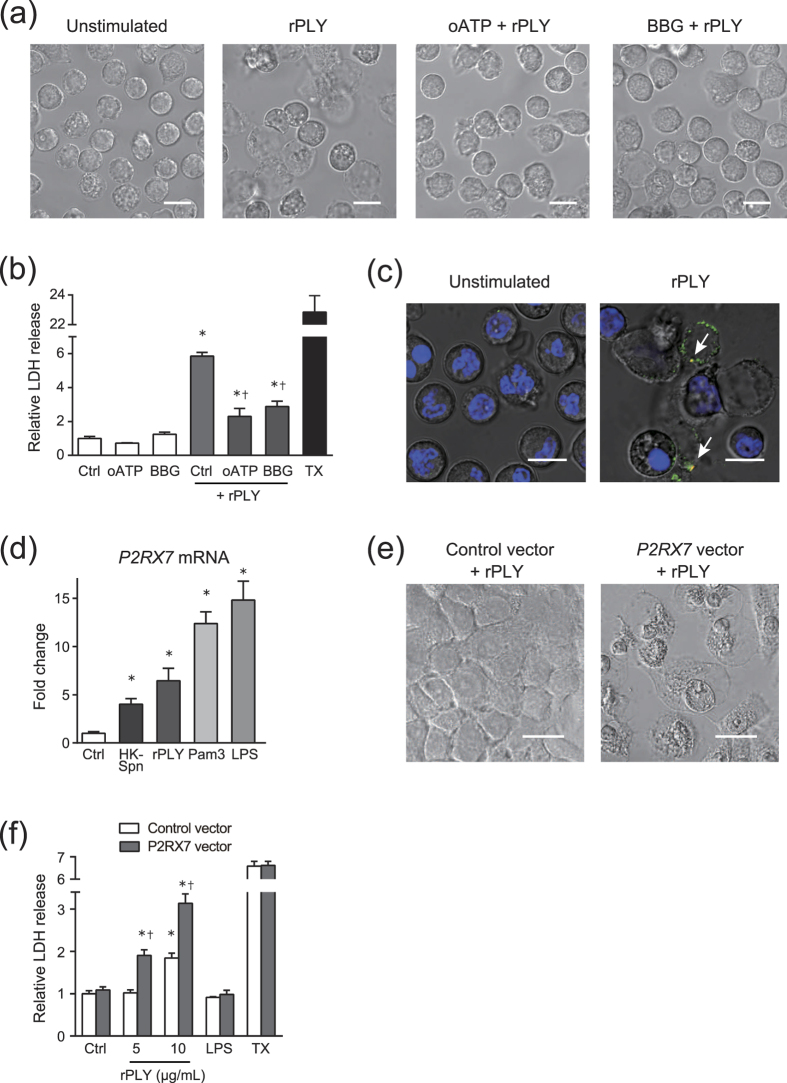
rPLY stimulation upregulates P2X_7_ receptor expression and induces cytotoxicity. (**a,b**) Neutrophils were pre-incubated for 1 h with P2X_7_ receptor antagonist (500 μM oxidized ATP and 3 μM BBG), followed by exposure to rPLY (5 μg/mL) for 3 h and (**a**) observation of morphologic changes in cells by optical microscope (scale bar: 10 μm). (**b**) An LDH assay was performed to evaluate cytotoxicity. (**c**) Representative fluorescence-microscopy images of rPLY-treated (5 μg/mL) neutrophils stained for DNA (DAPI; blue), P2X_7_ receptor (green), and rPLY (red). Arrows indicate co-localization of P2X_7_ receptor and rPLY (scale bar: 10 μm). (**d**) Real-time PCR was performed to quantify *P2RX7* mRNA in neutrophils stimulated with rPLY and TLR agonists for 3 h. The relative quantity of *P2RX7* mRNA was normalized to the relative quantity of *GAPDH* mRNA. (**e,f**) A549 cells were transfected with the *P2RX7* expression vector or empty vector, then stimulated with rPLY (5 μg/mL) for 6 h. (**e**) Morphologic changes in cells were observed by optical microscope (scale bar: 20 μm), and (**f**) an LDH assay was performed to evaluate cytotoxicity. Data represent the mean ± SD of quadruplicate experiments. (**b**) Data were evaluated using one-way analysis of variance with Tukey’s multiple-comparisons test. *Significantly different from the untreated-control group at *p* < 0.05. ^†^Significantly different from the rPLY-treated control group at *p* < 0.05. (**d**) Data were evaluated using one-way analysis of variance with Dunnett’s multiple-comparisons test. *Significantly different from the control group at *p* < 0.05. (**f**) Data were evaluated using one-way analysis of variance with Tukey’s multiple-comparisons test. *Significantly different at *p* < 0.05 from the untreated control within the same vector-transfected group. ^†^Significantly different at *p* < 0.05 from the control vector-transfected group using the same concentrations as those used in the rPLY-treated group. BBG, Brilliant Blue G; LDH, lactate dehydrogenase; NE, neutrophil elastase; rPLY, recombinant pneumolysin; SD, standard deviation; TX, Triton-X 100.

Previous studies have demonstrated that pore-forming toxins, such as alpha toxin from *Staphylococcus aureus* and beta toxin from *Clostridium perfringens,* produce cytotoxicity by specific interactions with the P2X_7_ receptor^24^,^25^. Therefore, we investigated whether rPLY stimulation induced P2X_7_ receptor-mediated cytotoxicity. As shown in Fig. 7a, pre-treatment of neutrophils with selective P2X_7_ receptor antagonists (oxidized ATP and Brilliant Blue G) inhibited PLY-induced cell lysis, although slight morphological alterations were observed. Additionally, these antagonists partially but significantly inhibited rPLY-induced LDH leakage from neutrophils (Fig. 7b). To confirm that rPLY was bound to the P2X_7_ receptor, we performed co-localization studies in rPLY-treated neutrophils subjected to immunostaining for rPLY and the endogenous P2X_7_ receptor. Our results indicated that rPLY co-localized with the P2X_7_ receptor at the cell surface (Fig. 7c). Additionally, unstimulated neutrophils rarely expressed the P2X_7_ receptor, whereas the P2X_7_ receptor was highly expressed in PLY-treated dead neutrophils. We considered that the expression level of the P2X_7_ receptor provided a mechanism to explain the higher sensitivity of neutrophils to rPLY treatment than that observed in A549 cells. Indeed, *P2RX7* mRNA was not detected in A549 cells (Fig. S5); however, *P2RX7* mRNA was detected in neutrophils, with upregulated levels of *P2RX7* transcription observed in the presence of heat-killed *S. pneumoniae,* TLR-agonist treatment, and rPLY stimulation (Fig. 7d). Figure S6 shows that rPLY stimulation did not significantly affect transcription of *TNFα* mRNA in neutrophils, suggesting that upregulation of *P2RX7* mRNA transcription in rPLY-treated neutrophils was not due to endotoxin contamination or TLR-dependent signalling. Furthermore, we observed that overexpression of P2X_7_ receptor in A549 cells enhanced rPLY-induced cell lysis (Fig. 7e), and that rPLY stimulation significantly increased LDH release from overexpressed A549 cells (Fig. 7f). These findings suggest that rPLY treatment elicited upregulation of P2X_7_ receptor expression and subsequent interaction between rPLY and P2X_7_ receptor triggered the cytotoxicity.

## Discussion

**Figure 8:**
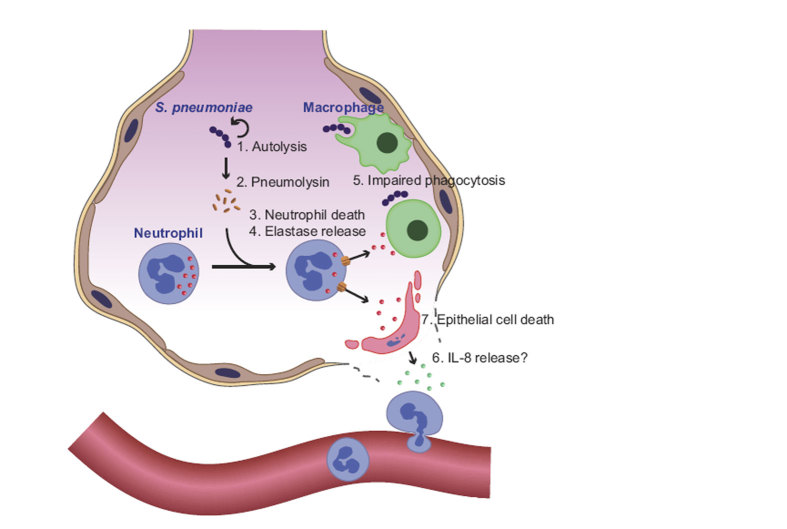
Summary of pneumococcus-induced lung injury. Pneumococcal LytA causes (1) autolysis of the bacteria and (2) release of PLY, which promotes pore formation in the neutrophil membrane and (3) induction of cell death. (4) NE is released by dead neutrophils and (5) impairs phagocytic activity of macrophages. Additionally, (6) NE may induce IL-8 production from alveolar epithelial cells. Finally, (7) NE triggers cell death, but does not exert bactericidal activity against pneumococcus. NE, neutrophil elastase; PLY, pneumolysin.

In this study, we showed that pneumococcal autolysis induces PLY release, which exerts cytotoxicity against infiltrating neutrophils through the P2X_7_ receptor. However, we also observed that PLY exhibited less cytotoxicity against alveolar epithelial cells and macrophages. Therefore, *S. pneumoniae* exploits NE leakage from degraded neutrophils to promote alveolar epithelial cell injury and impair the phagocytic function of macrophages (Fig. 8). Our findings demonstrated that pneumococcus may not simply attack lung tissue by its cytotoxic agent, but may also exploit host antimicrobial enzymes.

LytA is an enzyme that degrades the peptidoglycan backbone of bacterial organisms^26^, leading to bacterial autolysis. This enzyme is located in the cell envelope and is involved in a variety of other physiological cell functions^27^. We observed that, although treatment with Δ*lytA*-culture supernatant exhibited significantly less cytotoxicity than that observed following treatment with wild-type strain culture supernatant, administration of LytA itself did not induce cytotoxicity, suggesting that LytA-induced autolysis indirectly causes the release of bacterial virulence factors, including PLY. However, treatment with Δ*ply*-culture supernatant slightly but significantly induced cytotoxicity. These findings suggested that other cytotoxic agents might also be released by pneumococcal autolysis. One possible candidate molecule for this cytotoxicity is α-enolase, which is a pneumococcal glycolytic enzyme and induces neutrophil death with NETs formation^28^.

Although several reports demonstrated PLY cytotoxicity against alveolar epithelial cells, monocytes, T cells, and neutrophils^29^,^30^,^31^,^32^, there are no studies comparing the sensitivity of neutrophils with that of alveolar epithelial cells to PLY stimulation. Here, we observed that human neutrophils were more sensitive to PLY-induced cell lysis than A549 cells, and that excessive doses of PLY induced only minimal cytotoxicity in A549 cells. However, RAW264.7 cells were resistant to PLY-induced cell lysis. Although RAW264.7 cell may not accurately reflect characteristics associated with human macrophages, human monocyte-derived macrophages isolated from whole blood are also resistant to PLY-induced cell lysis^33^, which was consistent with our experimental findings. While no studies have so far reported PLY-induced cytolytic activity on alveolar macrophages, our findings suggested that infiltrated neutrophils are the primary target cells of PLY.

A major reason for the differences in cellular sensitivity to PLY-induced cytolysis is different cholesterol and phospholipid compositions of plasma membrane between various cell types^34^,^35^. It has been reported that liposomes composed of sphingomyelin in combination with artificially high concentration of cholesterol efficiently bind pneumolysin and other pore-forming toxins^36^. However, liposomes composed of cholesterol and other phospholipid had less binding affinity. Another reason may involve expression level of the purinergic receptor P2X_7_ on the cell surface^37^. Previous studies reported variations in cell sensitivity against the *C. perfringens*-produced pore-forming beta toxin, which induced cytotoxicity dependent upon expression levels of the P2X_7_ receptor in human immune-cell lines^25^. Here, neutrophils did not constitutively express the P2X_7_ receptor, and for this reason, pre-treatment with selective P2X_7_ receptor antagonists did not completely abolish rPLY-induced cell lysis. However, PLY stimulation significantly upregulated P2X_7_ receptor expression and induced cell lysis. Rather than performing P2X_7_ receptor knockdown experiments in neutrophils, we overexpressed human P2X_7_ receptor in A549 cells due to the short lifespan of neutrophils. Consistent with our findings in neutrophils and those of a previous study^25^, we observed increased cell-lysis activity in PLY-treated A549 cells overexpressing the P2X_7_ receptor. The mechanisms by which the P2X_7_ receptor is involved in pore-forming toxin-induced cell lysis are unclear; however, one possible mechanism involves the release of ATP by cells through PLY-induced membrane pores^38^, followed by ATP binding to P2X_7_ receptors, the subsequent opening of additional P2X_7_-associated pores, and cell lysis^37^.

Recently, several studies showed that NE inhibition reduced lung injury in animal models^17^,^18^. Interestingly, these studies also demonstrated that treatment with NE inhibitors reduced the number of viable pneumococci in infected lungs, suggesting that extracellular NE induces disruption of pulmonary immune defences rather than exerting bactericidal activity. Here, we observed that administration of ≥50 mU/mL and ≥125 mU/mL NE induced alveolar epithelial cell detachment and impaired the phagocytic function of macrophages, respectively, but did not affect pneumococcal viability. Additionally, NE concentrations are significantly elevated in bronchoalveolar-lavage fluid during severe pneumonia (average: 400 mU/mL)^15^. Therefore, it is likely that NE induces these events *in vivo*. Although the molecular mechanisms associated with NE-induced cell detachment are complex and not completely known, NE is thought to be involved in the disruption of cell-matrix adhesion and cell-cell junctions^39^,^40^. Furthermore, mechanisms associated with NE-induced inhibition of phagocytic activity may be related to cell rounding and detachment, because pseudopodia extension in activated macrophages is essential for phagocytosis and cell adherence^41^,^42^. Phagocytosis can also be controlled by a number of factors, including pro-inflammatory cytokines^43^, extracellular-matrix proteins, such as fibronectin^44^, or cell-surface molecules^45^. NE-induced proteolytic digestion of inflammatory cytokines and fibronectin may also inhibit macrophage activation^46^,^47^,^48^. Each of these NE-induced deleterious effects was ameliorated by administration of an NE inhibitor; therefore, controlling NE activity constitutes an attractive therapeutic technique for preventing lung injury in pneumococcal pneumonia.

In conclusion, our *in vitro* study demonstrated that pneumococcal pore-forming toxin was a major mediator of neutrophil cell death and subsequent release of NE, which eventually disrupted pulmonary immune defences. NE is exploited by pneumococcus as an etiological agent during pneumococcal pneumonia. From a therapeutic viewpoint and in addition to antibiotic use, novel strategies for disease treatment might include blockage of PLY infiltration by engineered liposomes^36^, neutrophil inhibition by chemokine-related antibodies or antagonists^49^,^50^, or NE inhibition by specific inhibitors^18^. Our findings also suggested that evolutionary progression of pneumococcus has allowed the release of PLY by self-destruction to exploit host molecules for promoting pneumococcal infection.

## Materials and Methods

### Bacterial strains and reagents

*S. pneumoniae* D39 (NCTC 7466) was purchased from the National Collection of Type Cultures (Salisbury, UK). *S. pneumoniae* Δ*lytA* was kindly provided by Dr. Shigetada Kawabata (Osaka University, Osaka, Japan)^46^. *S. pneumoniae* was grown in tryptic soy (TS) broth (Becton Dickinson, Franklin Lakes, NJ, USA) or 5% sheep-blood plates, with 100 μg/mL spectinomycin (Sigma-Aldrich, St. Louis, MO, USA) added to the medium to allow for *lytA*-negative mutant-strain selection. rLytA protein was kindly provided by Dr. Yuuki Sakaue (Niigata University, Niigata, Japan). Thiazolyl blue tetrazolium bromide (MTT) was purchased from Sigma-Aldrich, oxidized ATP was purchased from Merck Millipore (Billerica, MA, USA), and Brilliant Blue G was purchased from Abcam (Cambridge, UK). Mid-cell-anchored-protein Z antibody was generated by Eurofins Genomics K.K. (Tokyo, Japan).

### Construction of rPLY expression plasmid

The rPLY-expression plasmid was constructed using a pQE-30 vector (Qiagen, Hilden, Germany). The forward (5′- GGGGGATCCATGGCAAATAAAGCAGTAAA-3′) and reverse (5′- CCCCTCGAGCTAGTCATTTTCTACCTTATC-3′) primers were used to amplify the *ply* gene of *S. pneumoniae* D39 by polymerase chain reaction (PCR). The resulting PCR fragment was cloned into a pQE-30 vector, which was transformed into *Escherichia coli* strain M15 (Stratagene, San Diego, CA, USA) by electroporation using the Gene Pulser II Electroporation System (Bio-Rad Laboratories, Hercules, CA, USA). The M15 transformants were grown in Luria–Bertani broth (Nacalai Tesque, Kyoto, Japan) supplemented with 100 μg/mL ampicillin (Meiji Seika, Tokyo, Japan) to select for the pQE-30 vector and allowing its expression. The His-tagged rPLY protein was purified using a Ni-nitrilotriacetic acid column (Qiagen) under native conditions in accordance with the manufacturer’s instructions. The purified rPLY protein was dialyzed against phosphate buffered saline (PBS) and had a specific activity of 14,000 haemolytic units per mg of protein. A haemolytic unit is defined as the amount of rPLY contained in 1 ml of PBS which will cause 50% lysis of 1 ml of a 1% human erythrocyte suspension after 30 min at 37 °C. The amount of lipopolysaccharide (LPS) in 1 μg of purified rPLY protein was determined to be <2 pg according to a LPS-detection kit (GenScript, Piscataway Township, NJ, USA).

### Integration mutagenesis using a targeted plasmid

The PCR product of the internal portion of the *ply* gene was amplified using 5′-GGGGGATCCACCAACGACAGTCGCCTCTAG-3′ and 5′-CCCTCTAGATTCCATGCTGTGAGCCGTTAT-3′ primers and ligated into the suicide vector pSF151^51^. The resulting plasmid was transformed into wild-type strain D39 by adding 100 ng of plasmid and 100 ng of competence-stimulating peptide to 1 mL of exponentially growing cells at an optical density at 600 nm (OD_600_) of 0.1^52^. After incubation at 37 °C for 2 h, each culture was plated onto kanamycin (Meiji Seika)-containing blood-agar plates to select for the inactivated-mutant strain (Δ*ply*)^53^.

### Preparation of bacterial supernatant

*S. pneumoniae* strains were grown statically in TS broth at 37 °C for 20 h under aerobic conditions. Overnight cultures were inoculated into fresh TS broth to allow continued bacterial growth until reaching the exponential-growth phase (OD_600_ = 0.2). Cells were collected by centrifugation at 3000 *g* for 10 min and suspended in RPMI1640 medium, followed by an additional 12-h incubation. Thereafter, bacterial supernatants were collected by additional centrifugation at 3000 *g* for 10 min and subsequent filtration (0.22-μm filter; Merck Millipore) prior to their being used for assays.

### Human neutrophil isolation

Neutrophils were prepared as previously described^54^. Briefly, heparinized blood was obtained from healthy donors and mixed at a 1:1 ratio with PBS containing 3% dextran T500. After incubation at room temperature for 60 min, the supernatant was layered onto Ficoll-Paque (GE Healthcare, Little Chalfont, UK). After centrifugation at 450 *g* for 20 min, layers containing neutrophils were collected, and residual red-blood cells were lysed by hypotonic lysis. Viable cells were monitored using the Trypan-blue exclusion technique, and the cells were counted in a haemocytometer. The experimental protocol was approved by the Institutional Review Board of Niigata University, and the methods were carried out in accordance with the approved guidelines. Informed consent was obtained from all donors prior to their inclusion in this study (permit # 25-R9-07-13).

### Cell lines

Human alveolar epithelial cell line A549 (ATCC CCL-185) and mouse macrophage cell line RAW264.7 (ATCC TIB-71) were obtained from RIKEN Cell Bank (Ibaraki, Japan) and DS Pharma Biomedical Co. Ltd. (Osaka, Japan), respectively. These cell lines were grown in DMEM (Wako Pure Chemical Industries, Osaka, Japan) supplemented with 10% foetal bovine serum (Japan Bio Serum Co. Ltd., Hiroshima, Japan), 100 U/mL penicillin, and 100 μg/mL streptomycin (Wako Pure Chemical Industries) at 37 °C in 95% air and 5% CO_2_.

### Cytotoxicity assay

Human neutrophils (2 × 10^5^ in 200 μL) were seeded onto a 96-well plate (Becton Dickinson) and cultured in RPMI1640 medium containing 50% supernatant from *S. pneumoniae* D39, Δ*lytA,* or Δ*ply* cultures at 37 °C for 8 h. Cells were treated with Hoechst 33342 and ethidium homodimer III and visualised by a confocal laser-scanning microscope (Carl Zeiss, Jena, Germany). Also, Viable cells were determined using AlamarBlue cell-viability reagent (Thermo Fisher Scientific, Waltham, MA, USA) according to manufacturer instructions. Neutrophils, A549 cells (5 × 10^4^ in 200 μL), or RAW264.7 cells (5 × 10^4^ in 200 μL) were stimulated with various concentrations of rLytA (0.5–50 μg/mL), rPLY (0.25–10 μg/mL), LPS (100 ng/mL; Sigma-Aldrich), or heat-killed *S. pneumoniae* for 6 h. Additional cytotoxic analyses were performed using an LDH-measurement kit (Roche Diagnostics K.K., Tokyo, Japan).

### Western blot analysis

The supernatant from *S. pneumoniae* strains were mixed with 2% SDS-sample buffer, heated at 99 °C for 3 min, separated by SDS-PAGE using 12.5% gels (Gellex International, Tokyo, Japan), and transferred to polyvinylidene difluoride membranes (Merck Millipore). The membranes were incubated with blocking reagent (Nacalai Tesque) to block nonspecific binding and probed with the anti-PLY antibody (Abcam) diluted in Tris-buffered saline containing 0.05% Tween 20 (TaKaRa, Shiga, Japan). The membrane was then incubated with a HRP-conjugated secondary antibody (Cell Signalling Technologies, Danvers, MA, USA) in Tris-buffered saline containing 0.05% tween 20. The membrane was treated with HRP substrates (GE Healthcare) and analysed by chemiluminescence detector (Fujifilm, Tokyo, Japan). As a loading control, whole-cell lysates of *S. pneumoniae* strains prepared through the process of bacterial-supernatant preparation were immunoblotted with an antibody specific for pneumococcal mid-cell-anchored protein Z, which is a key membrane protein involved in bacterial-cell division and morphogenesis^55^.

### NE activity assay

Human neutrophils (5 × 10^6^ cells in 200 μL) were exposed to rPLY (5 μg/mL), Pam3CSK4 (1 μg/mL), LPS (100 ng/mL), or PMA (40 nM) for 8 h. NE activity in culture supernatant was evaluated using an elastase-activity assay kit (Cayman Chemical, Ann Arbor, MI, USA) according to manufacturer instructions.

### Quantitative real-time PCR

Gene expression in neutrophils or A549 cells was quantified using quantitative real-time PCR. Briefly, RNA was extracted from cell lysates using TRI Reagent (Molecular Research Center, Inc., Cincinnati, OH, USA) and quantified by spectrometry at 260 and 280 nm. The RNA was reverse transcribed using SuperScript VILO Master Mix (Thermo Fisher Scientific), and quantitative real-time PCR with cDNA was performed with the StepOnePlus real-time PCR system (Thermo Fisher Scientific) according to manufacturer protocol. TaqMan probes, sense primers, and antisense primers for expression of a housekeeping gene (*GAPDH*), NE (*ELANE*), or P2X_7_ receptor (*P2RX7*) were purchased from Thermo Fisher Scientific.

### Immunofluorescence analysis

rPLY-treated neutrophils were fixed and permeabilized using a cell fixation and permeabilization kit (Thermo Fisher Scientific) according to manufacturer instructions, followed by incubation of the cells in a blocking solution (Thermo Fisher Scientific) for 30 min. For NE detection, samples were stained with rabbit anti-NE antibody (Abcam) in the blocking solution. After overnight incubation at 4 °C, the secondary AlexaFluor 488-conjugated goat anti-rabbit IgG antibody (Thermo Fisher Scientific) in blocking buffer was added, followed by a 2-h incubation in the dark. For detection of the P2X_7_ receptor and PLY, samples were stained with goat anti-P2X_7_ receptor (Abcam) and mouse anti-PLY antibodies (Abcam). After overnight incubation at 4 °C, the secondary AlexaFluor 488-conjugated bovine anti-goat IgG antibody (Jackson ImmunoResearch, West Grove, PA, USA) was added, followed by incubation for 2 h. Samples were then washed with PBS and incubated with AlexaFluor 594-conjugated goat anti-mouse IgG antibody (Thermo Fisher Scientific) and observed with a confocal laser-scanning microscope (Carl Zeiss).

### Cell detachment assay

A549 and RAW264.7 cells (5 × 10^4^ cells in 200 μL) were cultured in serum-free DMEM containing various concentrations of NE (10–100 mU/mL and 125–1000 mU/mL, respectively; Innovative Research, Novi, MI, USA) or supernatant from rPLY-treated neutrophils in the presence or absence of the NE inhibitor sivelestat sodium hydrate (sivelestat, ONO-5046, N-[2-[4-(2,2-Dimethylpropionyloxy)phenylsulfonylamino]benzoyl] aminoacetic acid; ONO Pharmaceutical Co., Osaka, Japan) at 37 °C for 6 h. The cells were washed with DMEM to remove detached cells. Thereafter, MTT assays were performed to detect non-detached cells as previously described^56^.

### Phagocytic activity assay

*S. pneumoniae* D39 was cultured until reaching the exponential growth phase (OD_600_ = 0.2). RAW264.7 cells were cultured in serum-free DMEM and exposed to various concentration of NE (125–1000 mU/mL) or supernatant from rPLY-treated neutrophils in the presence or absence of the NE inhibitor sivelestat at 37 °C for 4 h. RAW264.7 cells were infected with *S. pneumoniae* (MOI = 10) for 1 h at 37 °C, followed by washing to remove extracellular non-adherent bacteria and a 1-h treatment with antibiotics (gentamicin [200 μg/mL] and penicillin [20 U/mL]) to eliminate residual or extracellular adherent bacteria. The macrophages were subsequently washed and lysed in sterile distilled water. Viable counts of phagocytized *S. pneumoniae* were determined by plating serially diluted macrophage lysates onto blood-agar plates subjecting them to aerobic culture.

For a quantitation of phagocytic activity, pHrodo red *S. aureus* bioparticles conjugate (Thermo Fisher Scientific), an indicator of phagocytic activity which increases in fluorescence as the pH of its surroundings, in Hank’s balanced-salt solution (Thermo Fisher Scientific) containing 20 mM HEPES (Nacalai Tesque) was added to each well containing NE-treated RAW264.7 cells, and the plate was incubated at 37 °C for 30 min. The cells stained with the indicator were counted by fluorescence microscopy (Keyence, Osaka, Japan), and fluorescence intensity per cell was calculated.

### Gene overexpression

A549 cells were transfected with the *P2RX7*-expression vector (Origene, Rockville, MD, USA) or empty vector using Lipofectamine 3000 reagent (Thermo Fisher Scientific) according to manufacturer protocol. Overexpression of *P2RX7* at the mRNA level was confirmed by quantitative PCR.

### Statistical analysis

Data were analysed statistically by one-way analysis of variance with Tukey’s or Dunnett’s multiple-comparisons test using Graph Pad Prism Software version 6.05 (GraphPad Software, Inc., La Jolla, CA, USA).

## Additional Information

**How to cite this article**: Domon, H. *et al*. *Streptococcus pneumoniae* disrupts pulmonary immune defence via elastase release following pneumolysin-dependent neutrophil lysis. *Sci. Rep.* **6**, 38013; doi: 10.1038/srep38013 (2016).

**Publisher's note:** Springer Nature remains neutral with regard to jurisdictional claims in published maps and institutional affiliations.

## Supplementary Material

Supplementary Dataset 1
