# Supplementary material for: Streptococcus pneumoniae disrupts pulmonary immune defence via elastase release following pneumolysin-dependent neutrophil lysis
Source: Sci Rep. 2016 Nov 28;6:38013. doi: 10.1038/srep38013 (PMC5125098; doi:10.1038/srep38013)
Supplement: Supplementary Dataset 1 [file srep38013-s1.doc]

Supplementary Information

***Streptococcus pneumoniae* disrupts pulmonary immune defence via elastase release following pneumolysin-dependent neutrophil lysis**

Hisanori Domon^1^, Masataka Oda^1^, Tomoki Maekawa^1,2^, Kosuke Nagai^1^, Wataru Takeda^3^, Yutaka Terao^1*^

^1^Division of Microbiology and Infectious Diseases, Niigata University Graduate School of Medical and Dental Sciences, Chuo-ku, Niigata, Japan

^2^Research Center for Advanced Oral Science, Niigata University, Graduate School of Medical and Dental Sciences, Chuo-ku, Niigata, Japan

^3^Faculty of Dentistry, Niigata University, Chuo-ku, Niigata, Japan

*Address correspondence to Yutaka Terao

Division of Microbiology and Infectious Diseases, Niigata University Graduate School of Medical and Dental Sciences, 2-5274, Gakkocho-dori, Chuo-ku, Niigata, 951-8514, Japan

Email: terao@dent.niigata-u.ac.jp


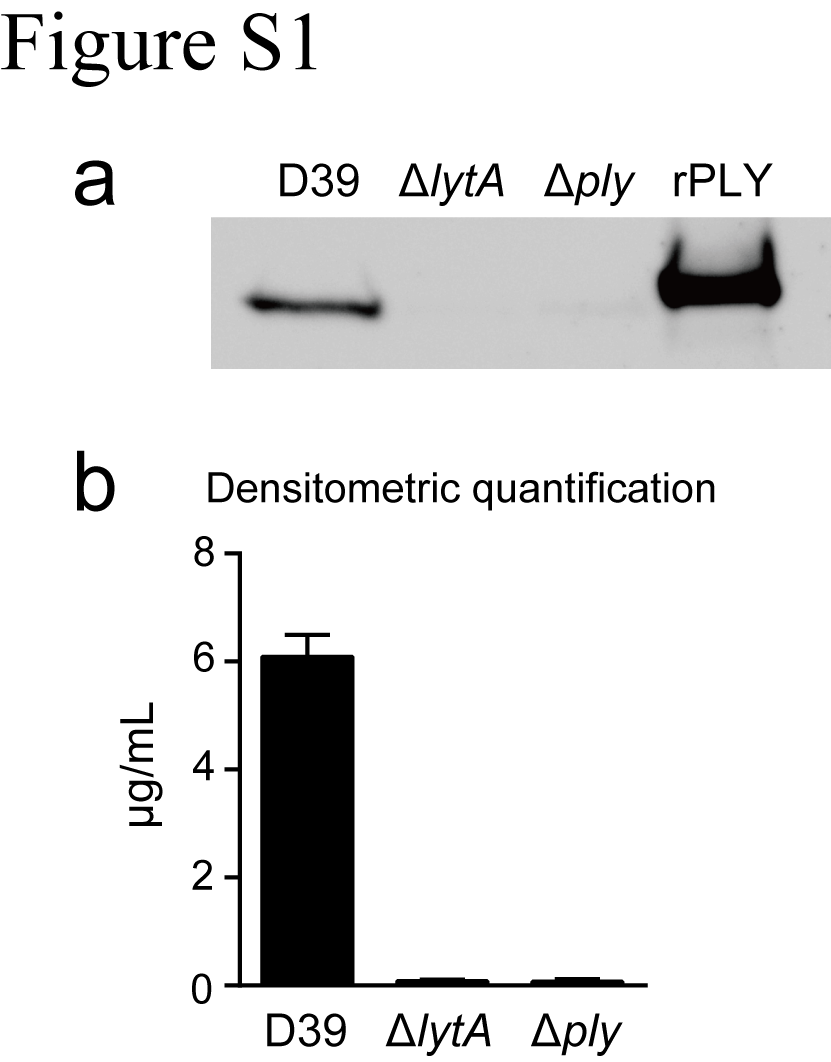


**Figure S1. Densitometric analysis revealed estimated concentration of PLY in *Streptococcus pneumoniae* culture supernatant.**

(a) Ten microliters of supernatant from *S. pneumoniae* D39 (wild-type), Δ*lytA*, or Δ*ply* cultures were separated by SDS-PAGE and immunoblotted using anti-PLY antibody. As a loading control, 250 ng of rPLY were also immunoblotted. The graph shows estimated concentration of PLY in these culture supernatant. (b) Densitometric analysis was performed by using Image Studio software ver. 3.1.4 (LI-COR Biotechnology, Lincoln, NE, USA). Data represent the mean ± SD of triplicate experiments.

rPLY, recombinant pneumolysin; SD, standard deviation.

**
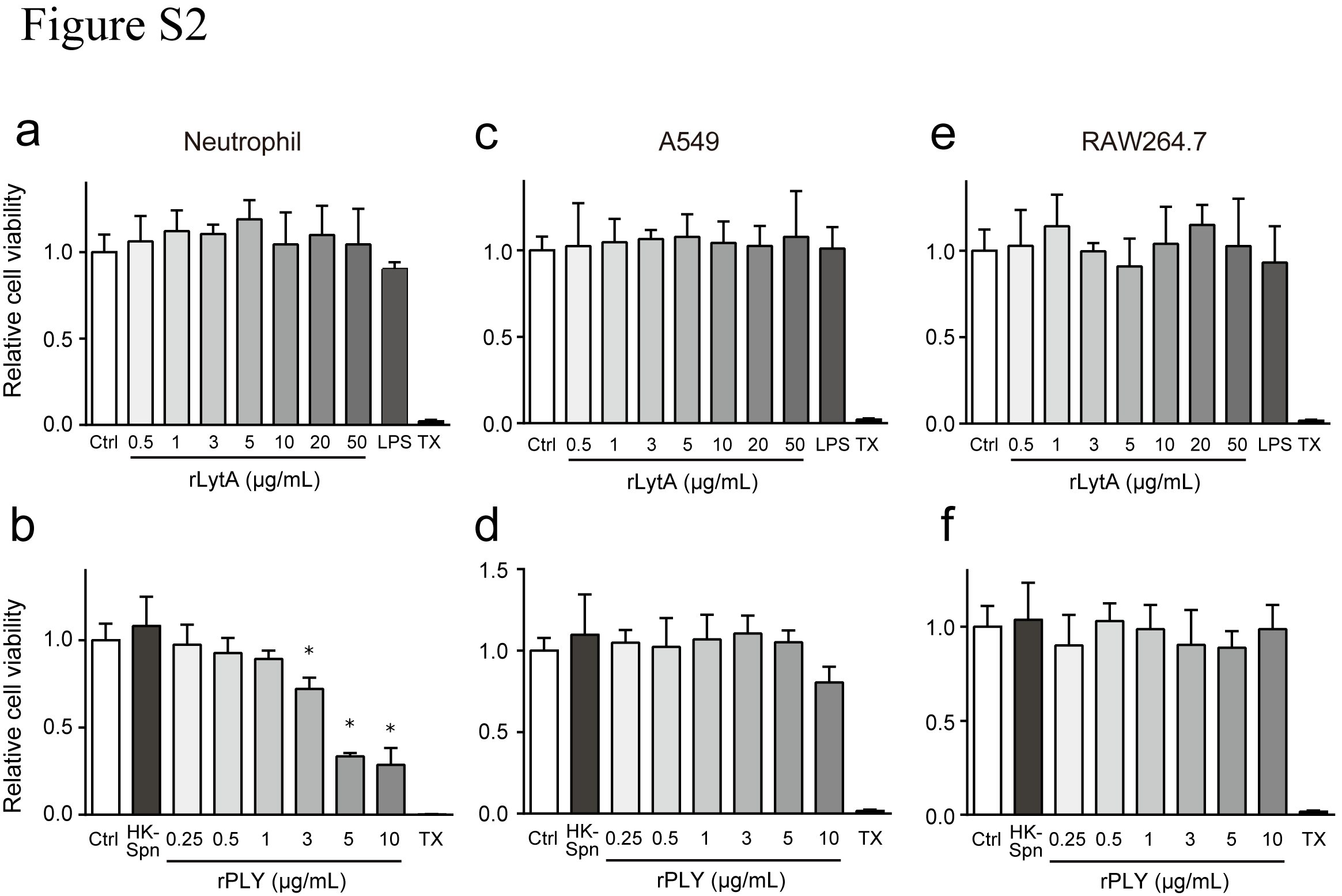
**

**Figure S2. rPLY decreased the number of viable neutrophils.**

(a and b) Human neutrophils, (c and d) A549 alveolar epithelial cells, or (e and f) RAW264.7 macrophages were exposed to various concentration of rLytA (0.5–50 μg/mL), LPS (100 ng/mL) (a, c, and e), rPLY (0.25–10 μg/mL), or heat-killed *Streptococcus pneumoniae* D39 (b, d, and f) for 6 h, followed by evaluation of AlamarBlue cell viability assay. Data represent the mean ± SD of quadruplicate experiments and were evaluated using one-way analysis of variance with Dunnett’s multiple-comparisons test. *significantly different from the control group at *p* < 0.05.

Ctrl, control; HK-Spn, heat-killed *Streptococcus pneumoniae* D39; LPS, lipopolysaccharide; rPLY, recombinant pneumolysin; SD, standard deviation; TX, Triton X-100.

**
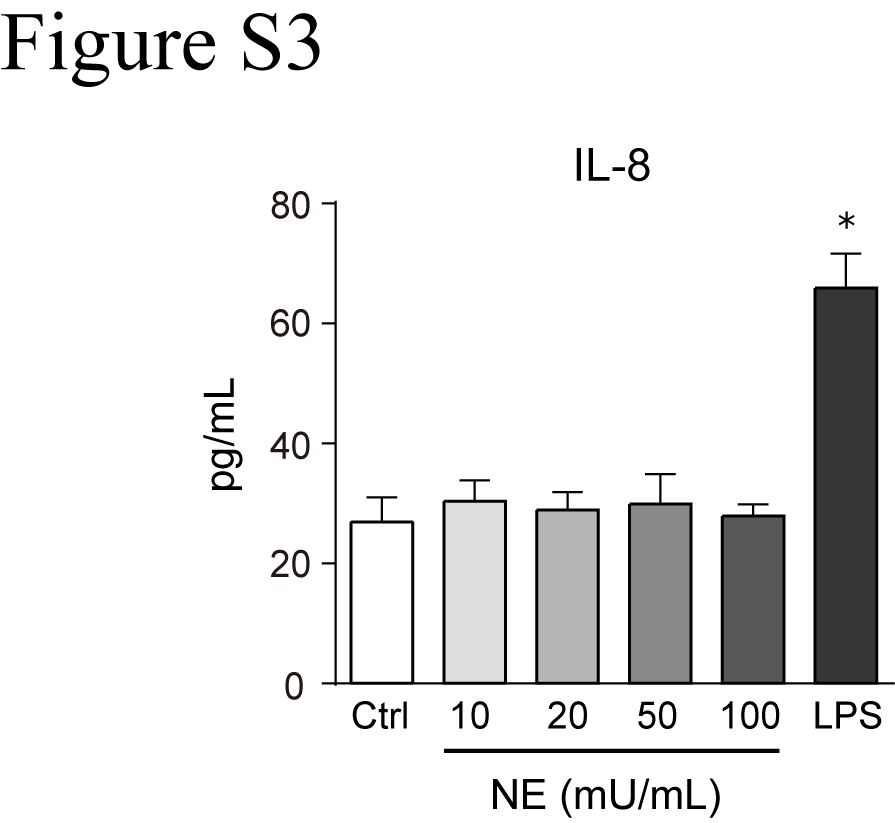
**

**Figure S3. IL-8 concentration was not enhanced in NE-treated A549 cells.**

A549 cells (5 × 10^4^ cells/200 μL) were cultured in serum-free DMEM and exposed to various concentrations (10–100 mU/mL) of NE or LPS (100 ng/mL) for 6 h and IL-8 concentration was analysed by ELISA. Data represent the mean ± SD of quadruplicate experiments and were evaluated using one-way analysis of variance with Dunnett’s multiple-comparisons test. *significantly different from the control group at *p* < 0.05.

Ctrl, control; NE, neutrophil elastase; SD, standard deviation.


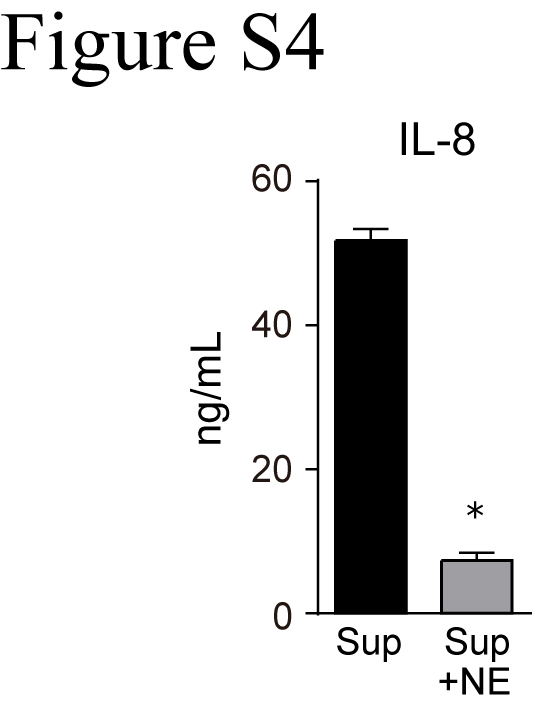


**Figure S4. NE degrades IL-8.**

The human monocytic cell line THP-1 (5 × 10^5^ cells/200 μL) was cultured in serum-free RPMI and exposed to 100 ng/mL of LPS for 6 h. Culture supernatant was harvested, mixed with 500 mU/mL NE, incubated at 37°C for 3 h, and IL-8 concentration was analysed by ELISA. Data represent the mean ± SD of quadruplicate experiments and were evaluated using non-paired Student *t* test. *significantly different from the control group at *p* < 0.05.

NE, neutrophil elastase; SD, standard deviation; Sup, culture supernatant.


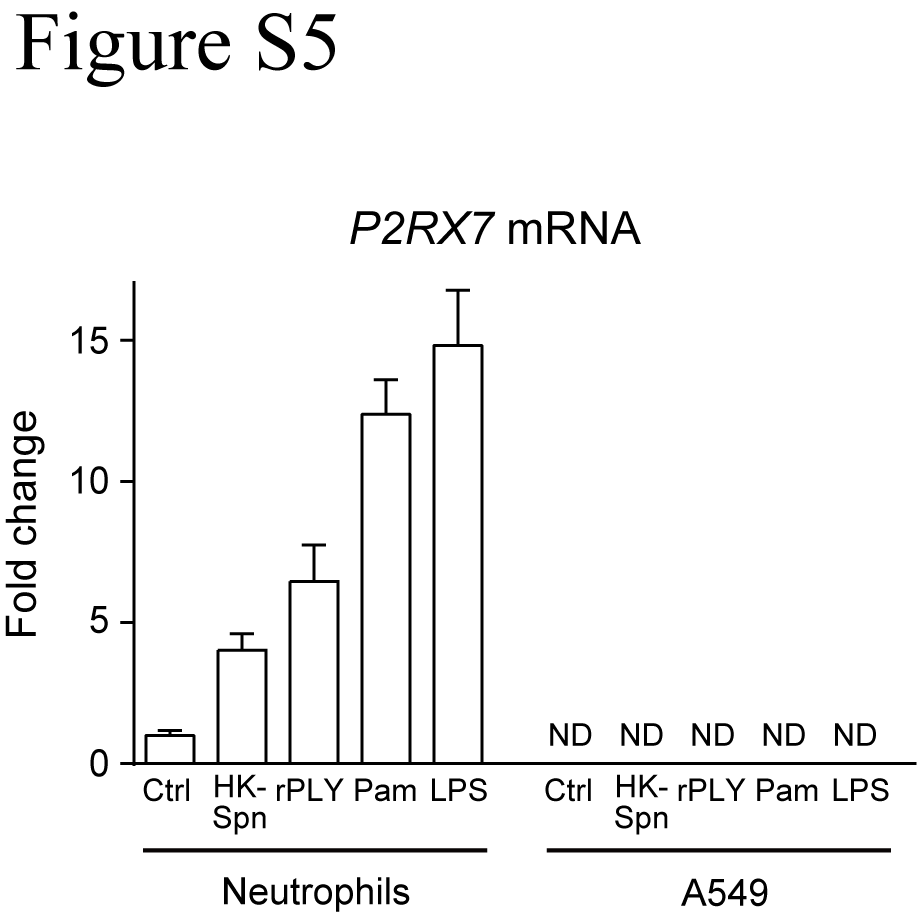


**Figure S5. *P2RX7* mRNA was not detected in A549 cells.** Neutrophils (2 × 10^6^ cells/500 μL) or A549 cells (3 × 10^5^ cells/500 μL) were exposed to heat-killed *Streptococcus pneumoniae* D39, rPLY (1 μg/mL), Pam3CSK4 (100 ng/mL), or LPS (10 ng/mL) for 3 h, followed by quantification of *P2RX7* mRNA by real-time PCR. The relative quantity of *P2RX7* mRNA was normalized against the relative quantity of *GAPDH* mRNA. Data represent the mean ± SD of quadruplicate experiments.

Ctrl, control, HK-Spn, heat-killed *Streptococcus pneumoniae* D39; Pam, Pam3CSK4; rPLY, recombinant pneumolysin; SD, standard deviation.


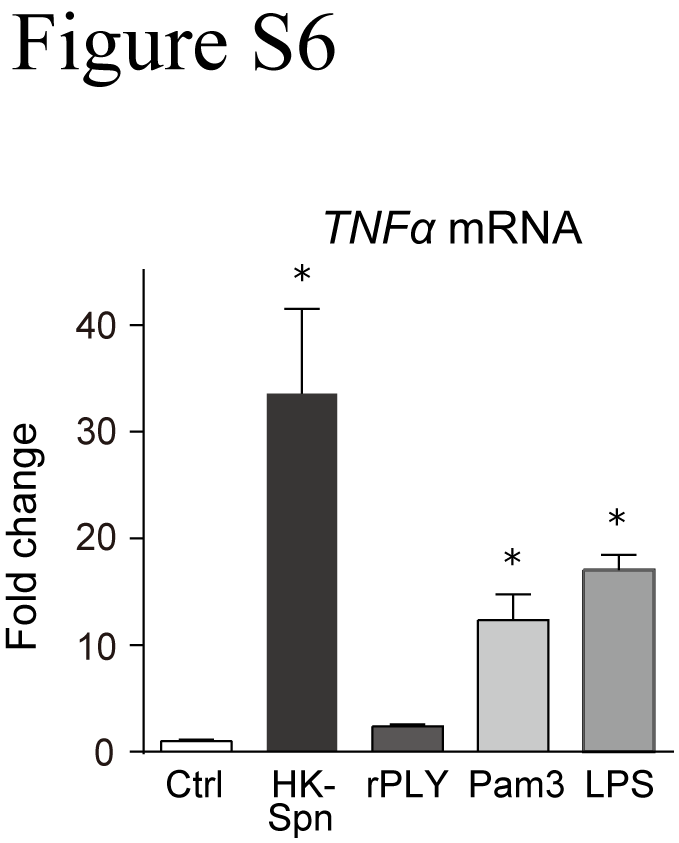


**Figure S6. rPLY stimulation did not significantly upregulate *TNFα* mRNA transcription in neutrophils.**

Neutrophils (2 × 10^6^ cells/500 μL) were exposed to heat-killed *Streptococcus pneumoniae* D39, rPLY (1 μg/mL), Pam3CSK4 (100 ng/mL), or LPS (10 ng/mL) for 3 h, followed by quantification of *TNFα* mRNA by real-time PCR. The relative quantity of *TNFα* mRNA was normalized against the relative quantity of *GAPDH* mRNA. Data represent the mean ± SD of quadruplicate experiments and were evaluated using one-way analysis of variance with Dunnett’s multiple-comparisons test. *significantly different from the control group at *p* < 0.05.

Ctrl, control, HK-Spn, heat-killed *Streptococcus pneumoniae* D39; Pam3, Pam3CSK4; rPLY, recombinant pneumolysin; SD, standard deviation.
